# Diagnosis of Cancer Spread Using Percutaneous Transhepatic Biliary Cholangioscopy-guided Ultrasonography for Malignant Bile Duct Stenosis

**DOI:** 10.1155/DTE.7.159

**Published:** 2001

**Authors:** Hirokazu Inoue

**Affiliations:** Third Department of Internal Medicine Toho University School of Medicine Ohashi Hospital 2-17-6, Ohashi, Meguro-ku Tokyo 153-8515 Japan

## Abstract

The characteristics of sites of intramural cancer spread were examined by comparing the intraductal ultrasonography (IDUS) and wall thickening findings at sites of intramural cancer spread and non-spread, in patients with malignant bile duct stenosis who had undergone percutaneous transhepatic biliary drainage (PTBD).

The subjects were ten patients with extrahepatic bile duct cancer, two with pancreatic cancer, one with cancer of the gallbladder, and one with cancer of the papilla of Vater who underwent preoperative IDUS. From these patients, 50 IDUS slices were examined with a congruent relationship with the histologic section of resected tissue. The maximum thickening, minimum thickening, maximum/minimum thickening ratio, and form factor of the medial and lateral margins of the medial hypoechoic layer were determined using diagnostic imaging, and the results were compared at sites of cancer spread and non-spread.

Twelve slices were obtained from the site of stenosis, 14 from sites of cancer spread, and 24 from non-spread sites. The maximum thickening, minimum thickening, and maximum/minimum thickening ratio differed significantly between the sites of spread and the non-spread.

The absolute values for wall thickening are useful for diagnosing the presence of intramural spread in patients with malignant biliary duct stenosis.


*Diagnostic and Therapeutic Endoscopy*, Vol. 7, pp. 159–164
Reprints available directly from the publisher
Photocopying permitted by license only

© 2001 OPA (Overseas Publishers Association) N.V.
Published by license under
the Harwood Academic Publishers imprint,
part of Gordon and Breach Publishing
a member of the Taylor & Francis Group.
All rights reserved.

### Diagnosis of Cancer Spread Using Percutaneous Transhepatic Biliary Cholangioscopy-guided Ultrasonography for Malignant Bile Duct Stenosis*

HIROKAZU INOUE†

*Third Department of Internal Medicine, Toho University School of Medicine, Ohashi Hospital, 2-17-6, Ohashi, Meguro-ku, Tokyo 153-8515, Japan*

The characteristics of sites of intramural cancer spread were examined by comparing the intraductal ultrasonography (IDUS) and wall thickening findings at sites of intramural cancer spread and non-spread, in patients with malignant bile duct stenosis who had undergone percutaneous transhepatic biliary drainage (PTBD).

The subjects were ten patients with extrahepatic bile duct cancer, two with pancreatic cancer, one with cancer of the gallbladder, and one with cancer of the papilla of Vater who underwent preoperative IDUS. From these patients, 50 IDUS slices were examined with a congruent relationship with the histologic section of resected tissue. The maximum thickening, minimum thickening, maximum/minimum thickening ratio, and form factor of the medial and lateral margins of the medial hypoechoic layer were determined using diagnostic imaging, and the results were compared at sites of cancer spread and non-spread.

Twelve slices were obtained from the site of stenosis, 14 from sites of cancer spread, and 24 from non-spread sites. The maximum thickening, minimum thickening, and maximum/minimum thickening ratio differed significantly between the sites of spread and the non-spread.

The absolute values for wall thickening are useful for diagnosing the presence of intramural spread in patients with malignant biliary duct stenosis.

*Keywords*: Intraductal ultrasonography; Malignant bile duct stenosis; Percutaneous transhepatic cholangioscopy; Cancer spread

### INTRODUCTION

Diagnosis of the longitudinal spread of extrahepatic bile duct cancer, particularly on the hepatic side, is important when selecting surgical procedures, such as resection margins. Intraductal ultrasonography (IDUS) using miniature ultrasonic probes is helpful for diagnosing longitudinal intramural spread [1], but in patients who have already undergone percutaneous transhepatic biliary drainage (PTBD), localization of the lesion is made difficult by inflammatory wall thickening caused by the drainage tube [2,3]. The

*A summary of this paper was presented at the 35th general meeting of the Japan Biliary Association (Hiroshima, October 1999).

†Corresponding author. Tel.: +81-3-3468-1251, ext. 3315. Fax: +81-3-3468-1269.

purpose of this study is to compare the IDUS and wall thickening findings at sites of intramural cancer spread and non-spread in patients with malignant bile duct stenosis who had previously undergone PTBD, and also to identify any differences between the two methods.

### PATIENTS AND METHODS

### Patients

The subjects were 14 patients who underwent preoperative IDUS, comprising of ten patients with extrahepatic bile duct cancer, two with pancreatic cancer, one with cancer of the gallbladder, and one with cancer of the papilla of Vater (Table I). From these patients, a total of 50 slices were studied at the porta hepatis, the ends of the stenosis, the central portion of the stenosis, the cystic duct bifurcation, and the upper margin of the intrapancreatic bile duct, at which sites, the ultrasound images and surgical specimens corresponded exactly.

IDUS images were excluded if no surgical specimen contained the corresponding site. Also, the sites that could not be delineated by ultrasound were excluded because the upper margin of the intrapancreatic bile duct and the cystic duct bifurcation were included in the lesion. Slices from the central portion of the stenosis, and on the duodenal side of this were excluded in two patients in whom the probe could not be advanced to the distal side due to the severity of stenosis.

### Test Methods

A CHF-T20 scope used for percutaneous transhepatic cholangioscopy (diameter of forceps' opening: 2.6 mm, Olympus Optical, Tokyo, Japan), a UM3R ultrasound probe (outer diameter of tip: 2.4 mm, maximum diameter: 2.5 mm, 20 MHz, Olympus Optical), and an EUM20 drive unit (Olympus Optical) were used. Endoscopy, fluoroscopy, and ultrasonography were simultaneously performed while observing the procedure on a television monitor. During cholangioscopy, the biliary tract was instilled with saline through the instrumentation channel, and it was ensured that the bile duct and the endoscopic image could be matched at all times while injecting contrast medium as required. The UM3R miniature ultrasonic probe was passed through the instrumentation channel of the CHF-T20 cholangioscope under endoscopic guidance, and IDUS was performed while watching the procedure on the monitor. The still ultrasound images were retained as appropriate, and the entire process was recorded on a video tape recorder.

### Image Analysis

The IDUS images were analyzed by feeding each image into an EM-1,2 two-dimensional manual measurement

TABLE I Subjects

| Patient no. | Anatomical regions | pTNM pathological classification | Stage grouping |
|---|---|---|---|
| 1. | Extrahepatic bile ducts | pT3N1M0 | IVA |
| 2. | Extrahepatic bile ducts | pT3N0M0 | IVA |
| 3. | Extrahepatic bile ducts | pT3N1M0 | IVA |
| 4. | Extrahepatic bile ducts | pT2N0M0 | II |
| 5. | Extrahepatic bile ducts | pT2N0M0 | II |
| 6. | Extrahepatic bile ducts | pT3N1M0 | IVA |
| 7. | Extrahepatic bile ducts | pT2N0M0 | II |
| 8. | Extrahepatic bile ducts | pT3N0M0 | IVA |
| 9. | Extrahepatic bile ducts | pT3N0M0 | IVA |
| 10. | Extrahepatic bile ducts | pT3N0M0 | IVA |
| 11. | Head of pancreas | pT2N1M0 | III |
| 12. | Head of pancreas | pT3N1M0 | III |
| 13. | Gallbladder | pT2N1M0 | III |
| 14. | Papilla of Vater | pT3N1M0 | III |

program (Rise, Miyagi, Japan), tracing the contours with a digitizer, and measuring the maximum and minimum thickening and the maximum/minimum thickening ratio of the medial hypoechoic layer in the slice (Fig. 1). The uniformity of the medial and lateral margins of the medial hypoechoic layer was also calculated using the formula for the form factor, an indicator of the degree of marginal unevenness (form factor = perimeter × perimeter/area × $1/4\pi$). The term "perimeter" means the lateral margin or medial margin in Fig. 1. The form factor for a circle is one and increases as the unevenness and deformity becomes more marked.

### Histological Diagnosis

The entire surgical specimen was cut at 5 mm intervals, and after determining the extent of cancer spread, hematoxylin and eosin stained specimens at the sites corresponding to the ultrasound images were examined again by microscopy to determine the presence or absence of cancer infiltration.

### Analysis of Measured Values

Fifty of the IDUS slices were to be compared with the tissue specimens. These were divided into three groups, comprising sites of cancer spread, non-spread sites, and the central portion of the stenosis, and the IDUS findings for each group were compared. The Mann–Whitney *U*-test was used to assess the significance of differences, and differences were considered to be significant, when $p < 0.05$.

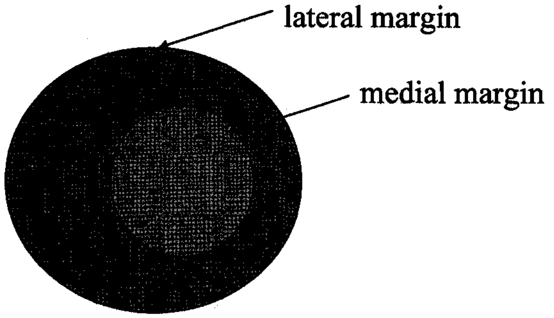

FIGURE 1 A frame from intraductal ultrasonography showed probe in the bile duct within ultrasonography field of view. A: maximum thickening of the medical hypoechoic layer; B: minimum thickening of the medial hypoechoic layer.

### RESULTS

The mean time between PTBD and IDUS was 23.4 days and ranged from 8 to 42 days. The 50 slices were divided into three groups based on the degree of histopathological spread in the corresponding specimens: sites of intramural spread (14 slices), sites of intramural non-spread (24 slices), and the central portion of the stenosis (12 slices) (Table II).

### Thickening of Medial Hypoechoic Layer

At sites of cancer spread, the mean maximum thickening was 3.4 mm (range: 1.5–5.3), the mean minimum thickening was 1.3 mm (0.5–2.2), and the mean maximum/minimum thickening ratio was 2.7 (1.1–4.5). At sites of non-spread, the mean maximum thickening was 1.7 mm, (0.7–2.9), the mean minimum thickening was 1.0 mm (0.5–2.1), and the mean maximum/minimum thickening ratio was 1.9 (1.3–3.3). At the site of stenosis, the mean maximum thickening was 4.4 mm (2.7–6.6), the mean minimum thickening was 1.6 mm (0.8–2.6), and the mean maximum/minimum thickening ratio was 3.0 (2.1–4.3). The maximum thickening, minimum thickening, and maximum/minimum thickening ratio, all differed significantly between the sites of cancer spread and non-spread (Table III).

### Uniformity of the Medial Hypoechoic Layer

At sites of cancer spread, the mean form factor was 1.76 (1.32–2.62) for the medial margin and 1.50 (1.21–1.73) for the lateral margin. At sites of non-spread, the mean form factor was 1.81 (1.31–4.15) for the medial margin and 1.57 (1.33–2.23) for the lateral margin. The mean form factor for the lateral margin at the site of stenosis was 1.83 (1.38–2.54). There were no significant differences between the sites of cancer

TABLE II Fifty slices of IDUS

| | No. of cases |
|---|---|
| Site of intramural spread: | |
| Positive | 14 |
| Negative | 24 |
| Central portion of the stenosis | 12 |

spread and non-spread with respect to the uniformity of either the medial or lateral margin (Table IV).

### Accuracy of Assessment of Cancer Spread Based on Wall Thickening

When cancer-spread-positive was defined in terms of the thickening of the medial hypoechoic layer, based on the fact that there was a difference between sites of cancer spread and non-spread with respect to the thickening of this layer, and the sensitivity and specificity were calculated per 0.1 mm, a thickening of 2 mm produced the best results. Overall, the sensitivity of a thickening of 2 mm for an accurate cancer-positive diagnosis was 85.7%, and the specificity was 66.7% (Table V).

### DISCUSSION

Diagnosis of the longitudinal spread of extrahepatic bile duct cancer on the hepatic side is important when deciding on surgical procedures, such as resection margins [4–6]. In a study of surgical bile duct cancer specimens, Shimada *et al.* noted that cancer spread on the hepatic side comprises superficial and intramural spread, and that they are not necessarily consistent. They found a mean 16.8 mm difference in the distance of spread between the two. In other words, superficial and intramural spreads need to be considered separately, when diagnosing cancer spread on the hepatic side. Cholangioscopy is useful for diagnosing superficial spread, and its diagnostic accuracy is improved, when combined with endoscopic fine needle biopsy [7]. However, endoscopy is only useful for diagnosing superficial spread; it does not provide an accurate indication of intramural spread. Intramural spread has traditionally been assessed using cholangiography. However, the accuracy of direct cholangiography for diagnosing spread on the hepatic side is limited. Taki *et al.* found that cholangiography was only 67% accurate for assessing the extent of cancer infiltration, even though the study was conducted retrospectively [8]. Recent studies have investigated the clinical application of miniature ultrasonic probes [9], and these are now used for diseases of the biliary tract. With these probes, IDUS can be used to examine the inside of the bile duct wall and to diagnose various biliary diseases [1,10,11]. Tamada *et al.* found that IDUS was more useful than direct cholangiography for diagnosing bile duct cancer spread on the hepatic side, with accuracies of 70 and 56%, respectively, [12].

The increasing focus on more detailed testing using IDUS has led to improvements, including the development of probes that can pass through the forceps' channel of endoscopes, the use of higher frequencies, and the development of models employing guide wires [13–15]. IDUS can be conducted using either a transpapillary approach or a percutaneous transhepatic approach. With the transpapillary approach, the probe is either inserted directly using the same technique by

TABLE III Thickness for medial hypoechoic layer

| | | A (mm) | B (mm) | A/B ratio |
|---|---|---|---|---|
| Site of intramural spread | Positive ($n = 14$) | 3.4 (1.5–5.3)* | 1.3 (0.5–2.2)** | 2.7 (1.1–4.5)* |
| | Negative ($n = 24$) | 1.7 (0.7–2.9)* | 1.0 (0.5–2.1)** | 1.9 (1.3–3.3)* |
| Central portion of the stenosis | ($n = 12$) | 4.4 (2.7–6.6) | 1.6 (0.8–2.6) | 3.0 (2.1–4.3) |

Values are expressed as mean (range:minimum–maximum).
$p < 0.01$ ** $p < 0.05$ by two-tailed Mann–Whitney *U*-test. A: maximum of the thickness for the medial hypoechoic layer; B: minimum of the thickness for the medial hypoechoic layer.

TABLE IV Form factor

| | | Medial margin | Lateral margin |
|---|---|---|---|
| Site of intramural spread | Positive ($n = 14$) | 1.76 (1.32–2.62) n.s. | 1.50 (1.21–1.73) n.s. |
| | Negative ($n = 24$) | 1.81 (1.31–4.15) n.s. | 1.57 (1.33–2.23) n.s. |
| Central portion of the stenosis | ($n = 12$) | | 1.83 (1.38–2.54) |

Values are expressed as mean (range:minimum–maximum).
Two-tailed Mann–Whitney *U*-test. n.s.: no significant difference.

TABLE V Diagnostic results by the thickness of the medical hypoechoic layer

| | | Site of intramural spread | |
|---|---|---|---|
| | | Positive | Negative |
| *All slices* ($n = 38$) | ≧2 mm | 13 | 7 |
| | <2 mm | 1 | 17 |
| | | Sensitivity 85.7% | Specificity 66.7% |

which the contrast medium tube is selectively inserted into the bile duct from the forceps' channel of the duodenoscope or by passing it over a guide wire retained in the bile duct [13–15]. Once inserted into the bile duct, the probe can be moved freely in and out in a vertical direction, but since the probe is maneuvered blindly, the region of interest cannot be scanned at will. Instead, it was decided to use the percutaneous transhepatic approach because the probe can be maneuvered more easily. The advantage of this approach is that the probe can be moved as desired because scanning can be performed under endoscopic guidance. In other words, the positional relationship between the lesion and the probe can be accurately determined, and slight adjustments can be made to the probe position by bending the cholangioscope. However, the percutaneous transhepatic approach requires forming a route of access for PTBD. The average time between the formation of a fistula from the puncture and IDUS was 23.4 days. Inflammatory wall thickening may occur if the tube is left in place for a prolonged period and frequently presents a problem for diagnostic imaging. Definite wall thickening usually occurs approximately 2 weeks after PTBD tube insertion [2,3] and is a problem because it is difficult to distinguish between wall thickening caused by cancer infiltration and inflammatory wall thickening.

The present study is conducted to determine whether inflammatory wall thickening caused by the PTBD tube might play a role in the diagnosis of longitudinal cancer spread in patients with malignant bile duct cancer. Other authors have noted that wall thickening caused by intramural cancer infiltration is unilateral and that the medial margin is often uneven [16–18]. Non-uniform wall thickening was quantified by measuring the maximum thickening, minimum thickening, and the maximum/minimum thickening ratio of the medial hypoechoic layer, and the results at sites of cancer spread and non-spread were compared. Findings revealed an appreciable significant difference in the maximum and minimum values between the two sites. The thickening of the medical hypoechoic layer (i.e. the wall thickening findings) is therefore an important factor in the diagnosis of cancer infiltration.

The uniformity of the medial and lateral margins of the medial hypoechoic layer was quantified by tracing the contours and calculating the form factor as an indicator of the extent of unevenness. However, it was not possible to discriminate based on margin configuration because there was no significant difference between the sites of cancer spread and non-spread. A previous study found that it was possible to discriminate histologically on the basis of the configuration of the medial and lateral margins [19], and from this study, it was noted that a non-uniform medial margin is associated with cancer infiltration. Therefore further studies using other quantitative analysis are needed.

Since the thickening of the medial hypoechoic layer is important in the diagnosis of cancer infiltration, as mentioned earlier, thickening was investigated and its diagnostic accuracy was calculated for cancer infiltration per 0.1 mm. The best results were obtained using a thickening of 2 mm (Table V), and the sensitivity and specificity using this value were found to be satisfactory. The fact that the specificity was lower than the sensitivity was probably due to a high false-positive rate, since a previous study reported a mean thickness of 2.0 mm for the medial hypoechoic layer of bile duct wall showing inflammatory thickening due to tube placement [2]. Thickening of the medial hypoechoic layer of 2 mm or greater that is continuous with the central portion of the stenosis should therefore be used as an indication of the extent of cancer infiltration in patients with malignant bile duct stenosis.

### CONCLUSIONS

This study shows that the absolute values for medial hypoechoic layer thickening are useful for diagnosing the presence of intramural cancer infiltration from IDUS images, and that thickening of the medial hypoechoic layer of 2 mm or greater, that is continuous with the central portion of the stenosis should be considered to represent the extent of cancer infiltration.

### *Acknowledgments*

I would like to thank Dr Masahiro Sato and Dr Yoshinori Igarashi of the Third Department of Internal Medicine at Toho University for their advice and Prof. Yoshihiro Sakai of the Third Department of Internal Medicine at Toho University for his supervision in the completion of this paper.

### *References*

[1] Tamada, K., Ido, K., Ueno, N., *et al.* (1995) "Preoperative staging of extrahepatic bile duct cancer with intraductal ultrasonography", *Am. J. Gastroenterol.* **90**, 239–246.
[2] Tamada, K., Tomiyama, T., Ichiyma, M., *et al.* (1998) "Influence of biliary drainage catheter on bile duct wall thickness as measured by intraductal ultrasonography", *Gastrointest. Endosc.* **47**, 28–32.
[3] Noda, Y., Fujita, N., Kobayashi, G., *et al.* (1994) "Endoscopic ultrasonography (EUS) in the assessment of the extent of bile duct cancer", *J. Bil. Panc.* **15**, 1029–1035, (in Japanese).
[4] Shimada, H., Nimoto, S., Matsuba, A., *et al.* (1988) "The infiltration of bile duct carcinoma along the bile duct wall", *Int. Surg.* **73**, 87–90.
[5] Yamaguchi, K. (1992) "Macroscopic types and mode of extension of extrahepatic bile duct carcinomas", *Endoscopia Digestiva* **4**, 353–358, (in Japanese with English abstract).
[6] Hayashi, S., Miyazaki, M., Kondou, Y., *et al.* (1994) "Invasive growth patterns of hepatic hilar ductal carcinoma", *Cancer* **73**, 2922–2929.
[7] Sato, M., Inoue, H., Ogawa, S., *et al.* (1998) "Limitations of percutaneous transhepatic cholangioscopy for the diagnosis of the intramural extension of bile duct carcinoma", *Endoscopy* **30**, 281–288.
[8] Ryu, M., Uematsu, S., Watanabe, Y., *et al.* (1980) "The study on surgical procedure for upper bile duct carcinoma investigating from radiological findings", *J. Jpn. Surg. Soc.* **81**, 782–790.
[9] Silverstein, F.E., Martin, R.W., Kimmey, M.B., *et al.* (1989) "Experimental evaluation of an endoscopic ultrasound probe: *in vitro* and *in vivo* canine studies", *Gastroenterology* **96**, 1058–1062.
[10] Noda, T., Ido, K., Ueno, N., *et al.* (1995) "Transpapillary intraductal ultrasonography of the bile duct without sphincterotomy", *Ultrasound Int.* **1**, 141–147.
[11] Tamada, K., Ueno, N., Tomiyama, T., *et al.* (1998) "Characterization of biliary structure using intraductal ultrasonography comparison with percutaneous cholangioscopic biopsy", *Gastrointest. Endosc.* **47**, 341–349.
[12] Tamada, K., Ueno, N., Tomiyama, T., *et al.* (1997) "Assessment of tumor extension and therapeutic plan for bile duct cancer using the information from intraductal ultrasonography", *Endoscopia Digestiva* **9**, 639–652, (in Japanese).
[13] Mukai, H., Konishi, J., Ikeda, E., *et al.* (1991) "Clinical evaluation of the ultrasonic probe in the diagnosis of biliary and pancreatic diseases", *Gastroenterol. Endosc.* **33**, 519–526, (in Japanese with English abstract).
[14] Fujita, N., Noda, Y., Kobayashi, G., *et al.* (1993) "Transpapillary biliary sonography with a microscanner", *Gastroenterol. Endosc.* **35**, 2680–2686, (in Japanese with English abstract).
[15] Tamada, K., Ueno, N., Ichiyama, M., *et al.* (1995) "Assessement of tumor extension in bile duct cancer using intraductal ultrasonography", *Diagn. Imaging Abdom.* **15**, 947–962, (in Japanese).
[16] Yamanaka, T. (1992) "Fundamental and clinical studies of ultrasonic microprobe in biliary tract", *Gastroenterol. Endosc.* **34**, 1601–1610, (in Japanese with English abstract).
[17] Asahara, S., Ariyama, J., Suyama, M., *et al.* (1995) "Evaluation of tumor extension of extrahepatic bile duct carcinoma by intraductal ultrasonography", *Endoscopia Digestiva* **7**, 1361–1368, (in Japanese with English abstract).
[18] Noda, Y., Fujita, N., Kobayashi, G., *et al.* (2000) "Intraductal ultrasonography (IDUS) for bile duct cancer using a wire-guided ultrasonic probe", *Endoscopia Digestiva* **12**, 830–831, (in Japanese).
[19] Tamada, K., Kanai, N., Tomiyama, T., *et al.* (1999) "Prediction of the histologic type of bile duct cancer by using intraductal ultrasonography", *Abdom. Imaging* **24**, 484–490.

